# Health-Related Quality of Life in Spinal Muscular Atrophy Patients and Their Caregivers—A Prospective, Cross-Sectional, Multi-Center Analysis

**DOI:** 10.3390/brainsci13010110

**Published:** 2023-01-07

**Authors:** Camilla Wohnrade, Ann-Kathrin Velling, Lucas Mix, Claudia D. Wurster, Isabell Cordts, Benjamin Stolte, Daniel Zeller, Zeljko Uzelac, Sophia Platen, Tim Hagenacker, Marcus Deschauer, Paul Lingor, Albert C. Ludolph, Dorothée Lulé, Susanne Petri, Alma Osmanovic, Olivia Schreiber-Katz

**Affiliations:** 1 Department of Neurology, Hannover Medical School, 30625 Hannover, Germany; 2 Department of Neurology, University of Ulm, 89081 Ulm, Germany; 3 Department of Neurology, Klinikum rechts der Isar, Technical University of Munich, 81675 Munich, Germany; 4 Department of Neurology and Center for Translational Neuro- and Behavioral Science, University Medicine Essen, 45147 Essen, Germany; 5 Department of Neurology, University of Wuerzburg, 97080 Wuerzburg, Germany; 6 German Center for Neurodegenerative Diseases, 89081 Ulm, Germany; 7 Essen Center for Rare Diseases (EZSE), University Hospital Essen, 45147 Essen, Germany

**Keywords:** caregiver, caregiver burden, mental health, quality of life, spinal muscular atrophy, patient reported outcome measures

## Abstract

Spinal muscular atrophy (SMA) is a disabling disease that affects not only the patient’s health-related quality of life (HRQoL), but also causes a high caregiver burden (CGB). The aim of this study was to evaluate HRQoL, CGB, and their predictors in SMA. In two prospective, cross-sectional, and multi-center studies, SMA patients (*n* = 39) and SMA patient/caregiver couples (*n* = 49) filled in the EuroQoL Five Dimension Five Level Scale (EQ-5D-5L) and the Short Form Health Survey 36 (SF-36). Caregivers (CGs) additionally answered the Zarit Burden Interview (ZBI) and the Hospital Anxiety and Depression Scale (HADS). Patients were clustered into two groups with either low or high HRQoL (EQ-5D-5L index value <0.259 or >0.679). The latter group was mostly composed of ambulatory type III patients with higher motor/functional scores. More severely affected patients reported low physical functioning but good mental health and vitality. The CGB (mean ZBI = 22/88) correlated negatively with patients’ motor/functional scores and age. Higher CGB was associated with a lower HRQoL, higher depression and anxiety, and more health impairments of the CGs. We conclude that patient and CG well-being levels interact closely, which highlights the need to consider the health of both parties while evaluating novel treatments.

## 1. Introduction

Spinal muscular atrophy (SMA) is a neuromuscular disease (NMD) frequently caused by homozygous or compound heterozygous deletion of exons 7 and 8 of the *survival motor neuron* (*SMN*) *1* gene on chromosome 5q13 [1]. Lack of SMN protein results in motor neuron loss and subsequent muscular atrophy, progressive paralysis, dysphagia and respiratory insufficiency, and complications such as scoliosis and contractures. Traditionally, different SMA types can be distinguished according to the time of symptom onset and the best-achieved motor milestones during child development [2]. Types I and II manifest earlier in life (zero to six months after birth vs. seven to 18 months) than types III and IV (adult subtype, onset age > 30 years), and if untreated, lead to early physical disability and drastically reduced life expectancy. SMA type III manifests after the age of 18 months with a more variable disease course and often normal life expectancy though severe impairment [3]. Disease severity correlates inversely with the number of copies of the *SMN2* gene, a nearly identical copy of *SMN1* that can partly rescue the survival of motor neurons [4,5,6,7]. Since the introduction of new therapeutic options, these phenotypes are evolving. Nevertheless, all patients need intense, multidisciplinary treatment and are highly dependent on medical, therapeutic, and care resources [8].

The antisense oligonucleotide (ASO) nusinersen as first causative gene-modifying treatment for SMA [9,10] can improve or at least stabilize motor function in adult SMA patients [11,12,13], but only few studies have evaluated its impact on patient-reported outcome measures (PROM) such as health-related quality of life (HRQoL) [14,15]. We hypothesize that HRQoL could be an important additional outcome measure [16,17], as motor scores have a reduced significance in severely affected patients due to floor effects. Therefore, HRQoL needs to be characterized thoroughly in this population.

Another often neglected but important aspect of NMDs is the caregiver burden (CGB) of informal caregivers, who provide unpaid care to someone with whom they have a personal relationship (most commonly a family member). It has been shown that CGB in neurological diseases is high and associated with disease severity, the patient’s dependence on the caregiver (CG), the patient’s age, and the relationship between the patient and the CG, as well as the amount of care the CG has to provide [18,19]. CGB affects the CGs’ physical and mental health as well as their HRQoL. Different predictors for lower HRQoL and health impairments of the CG, such as the presence of executive dysfunction in the patient or mental attitude of the CG, which are concomitant with a higher burden, have been evaluated in various neurological diseases [20,21,22,23]. However, there is little information on CGB and its effects on the CG in SMA.

Therefore, the aims of this study were to characterize HRQoL in patients with SMA and their CGs and to investigate the informal CGB, its influencing factors, and its consequences for CGs in SMA.

## 2. Materials and Methods

### 2.1. Study Design and Setting

The data incorporated in this manuscript were obtained in two prospective cross-sectional multi-center studies. Participants were recruited at five specialized motor neuron disease clinics, which are members of the MND Net (German Network for motor neuron diseases) [24]: Hannover (principal investigator); Ulm; Munich; Wuerzburg; and Essen. Between June 2018 and January 2021, 5q-associated SMA patients were recruited at Hannover and Ulm sites for the evaluation of their HRQoL. In a second study, couples consisting of a 5q-associated SMA patient and his/her CG were recruited in all five centers from November 2018 to March 2020.

All participants were approached during routine outpatient or inpatient visits. They were offered the opportunity to complete a paper-based questionnaire during their visit and gave their written informed consent for the use of their pseudonymized data.

This study report was structured following the reporting guidelines of Strengthening the Reporting of Observational Studies in Epidemiology (STROBE) [25].

### 2.2. Participants

Inclusion criteria comprised a genetically confirmed 5q-associated SMA diagnosis of the patient, the ability to speak and understand German, and an age of ≥18 years as well as the ability to complete the study questionnaire at least with the help of a proxy. Cognitive impairment preventing completion of the questionnaire was an exclusion criterion. In order to avoid a selection bias, all patients and patient/CG couples were asked to participate if they met the inclusion criteria and visited one of the clinics listed above for treatment during the data enquiry period.

For the analysis of HRQoL, only SMA patients who underwent nusinersen loading during the recruitment period were approached. In total, *n* = 48 patients with 5q-SMA at the Hannover (*n* = 34) and Ulm (*n* = 14) sites were potentially eligible and confirmed eligible. One patient at the Hannover site declined study participation (reason not specified), leaving *n* = 47 participants included in the study. *n* = 8 patients at the Hannover site had incomplete baseline data so that datasets of *n* = 39 patients were analyzed. The EQ-5D-5L was assessed at the Hannover site only (*n* = 22).

Moreover, *n* = 49 patient/CG couples were identified at the individual sites (Ulm *n* = 17, Hannover *n* = 14, Munich *n* = 9, Essen *n* = 5, Wuerzburg *n* = 4) and included in the analysis of CGB and HRQoL in CGs. Of the potentially/confirmed eligible patient/caregiver couples, some were ruled out due to simultaneous participation in an interventional study at certain sites. Of the patient/caregiver couples approached, one at the Hannover site declined participation in this study (reason not specified). Finally, two distinct but overlapping study populations were recruited for the evaluation of HRQoL in SMA patients and the evaluation of HRQoL in the CGs/CGB.

### 2.3. Assessment Instruments

#### 2.3.1. Patient Questionnaires and Assessments

During the nusinersen-loading period, the patients filled in a self-reported questionnaire, and physiotherapists or study nurses assessed the patients’ motor function using the Hammersmith Functional Motor Scale Expanded (HFMSE) and the Revised Upper Limp Module (RULM), as they are validated and used most commonly in SMA patients [26,27]. Due to the lack of validated disease-specific tools for the assessment of activities of daily living (ADL) in SMA patients, the revised Amyotrophic Lateral Sclerosis Functional Rating Scale (ALSFRS-R) and the Barthel-Index (BI) were assessed in an interview by trained raters (see Section 2.3.3). The first part of the self-reported questionnaire was a self-designed overview of the patient’s demographics (diagnosis, gender, age, body mass index, marital status, federal state, educational years) and disease history (nature of first symptoms, age at disease onset, genetics, age at therapy start). The second part consisted of a medical assessment (nutrition, pulmonary function, orthopedics, hospitalization, medication, motor function, wheelchair use (without specification of the amount of time spent in the wheelchair), findings in the clinical examination and adverse events). The third part included a battery of PROM: among others, the Short Form Health Survey 36 (SF-36), the EuroQoL Five Dimension Five Level Scale (EQ-5D-5L) [28], and the twelve-item Amyotrophic Lateral Sclerosis Depression Inventory (ADI-12) were included and are reported in the present study. Both, the SF-36 and the EQ-5D-5L are widely spread generic measures to assess HRQoL. Specific tools for MND patients, such as the Individualized Neuromuscular Quality of Life (INQoL) [29], and data published about these tools are scarce. To be able to compare the data collected in this study with previous studies, we decided to use the above-mentioned generic tools.

#### 2.3.2. Caregiver Questionnaire

CGs filled in the study questionnaire at one random time-point during ongoing nusinersen treatment of the patient they cared for. The first part of the CG questionnaire inquired after the baseline demographics of the CG as well as the patient (gender, age, diagnosis, marital status, relationship to the patient, educational years). In the second part, the patient’s disease history and motor abilities (age at disease onset and therapy start, wheelchair use, percutaneous endoscopic gastrostomy (PEG), non-invasive ventilation (NIV), CG-rating of current BI) were assessed. Furthermore, the CGs stated their employment status, the duration of care (DOC) in hours that they provided per day, nursing precautions (necessity of permanent attendance of the CG) and their own health impairments incurred as a result of caregiving. In the last part, CGs filled in the Zarit-Burden Interview (ZBI), the SF-36, EQ-5D-5L, and the Hospital Anxiety and Depression Scale (HADS).

#### 2.3.3. Motor Function and Daily Activities

The HFMSE is a disease-specific scale commonly used in SMA type II and III patients to measure gross motor function. It has 33 items, which are scaled with two points if the patient is able to perform the task unaided, one point if the patient is able with assistance, or zero points if the patient is unable to perform the activity. A sum-score is calculated with a maximum of 66 points, which indicates that all activities are possible without help [26,30,31].

The RULM measures upper limb function in SMA. It consists of 20 items, which are subdivided into 1 entry item and 19 items testing various motions, each scored on a three-point scale from zero points (unable) to one point (able, with modification) to two points (no difficulty). The maximum sum-score is 37 points, which implies that all tasks can be exercised without difficulty [27,32].

The ALSFRS-R was designed to capture the impairment in the daily routine of patients with amyotrophic lateral sclerosis (ALS). It consists of twelve items, which subdivide into four dimensions: gross motor function, fine motor function, bulbar function and respiratory function. The patients are asked to assess their functioning on a five-point scale from four (no loss of function) to zero (total loss of function). The sum-score ranges from zero to 48 points, indicating the level of impairment [33]. The ALSFRS-R is suitable for use with SMA patients and applied frequently in SMA patients [13,34,35,36], but has not been validated for SMA up to now.

Additionally, the BI was assessed to measure performance in ADL. It consists of ten questions, which include the activities of feeding, bathing, grooming, dressing, bowel and bladder care, toileting, bed and chair transfer, general mobility, and climbing stairs. The performance in each activity is rated with zero, five or ten (and for some activities up to fifteen) points. Summed up, the scale ranges between 0 and 100 points [37,38].

#### 2.3.4. Heath-Related Quality of Life (HRQoL)

The EQ-5D-5L is a validated self-reported instrument to measure HRQoL. It involves five different dimensions: mobility, self-care, usual activities, pain/discomfort, and anxiety/depression. Each dimension is scored in a single item from one (no problems) to five (severe problems). Taken together, the scores for all dimensions result in different health states from 11,111 (best health) to 55,555 (worst health). This health state can be converted into an index value that ranges from −0.661 (worst) to 1 (best health), using a value set derived from a country-specific (German) reference sample [39,40]. In addition, patients are asked to score their health state “today” on a Visual Analog Scale (VAS), which ranges from 0 (worst possible health) to 100 (best possible health). The EQ-5D-5L is widely used for different diseases as well as for healthy individuals to evaluate and compare quality of life [41,42].

The second scale used to evaluate quality of life was the Short Form 36 Health Survey (SF-36), which is a self-reported questionnaire referring to the individual’s health state within the last four weeks. It measures health in eight multi-item dimensions: Physical Functioning (PF), Role Physical (RP), Bodily Pain (BP), General Health (GH), Vitality (VT), Social Functioning (SF), Role Emotional (RE) and Mental Health (MH). The 36 items are assessed on a five- or six-point Likert scale and the scores within each dimension are converted into a standardized sum-score ranging from 0 (worst health) to 100 (best health). Additionally, a ninth one-item dimension (Health Transition (HT)) exists, which is scaled ordinally with 0, 25, 50, 75, or 100 points. Though it has not been validated in the SMA population, the factor structure of the SF-36 was replicated in other MNDs [43,44,45,46].

#### 2.3.5. Depression and Anxiety

Patients’ depression was assessed using the ADI-12, an adaptation of the Beck`s depression inventory, which was originally designed for ALS patients discarding all possibly MND-related symptoms. The inventory consists of twelve items scored on a four-point Likert type scale, where the patients are asked to review the last two weeks. The calculated sum-score ranges from 0 (best possible) to 48 (worst possible). Scores between 22 to 28 are considered a mild depression. Scores > 28 are generally considered as clinically relevant depression [47].

The HADS is a self-report instrument to assess anxiety (HADS-A) and depression (HADS-D) in patients attending non-psychiatric clinics and is commonly used in studies evaluating CGs/CGB [48,49]. It consists of 14 items, which can be ranked on a four-point scale (zero to three). There is an anxiety subscale and a depression subscale, which consist of seven items each. The range for each subscale is from 0 to 21 points, adding up to a maximum score of 42. The cut-off for being at risk of an anxiety disorder or depression has been established at eight points for each subscale in previous studies [50,51].

#### 2.3.6. Caregiver Burden

CGB was measured using the ZBI, a structured interview that ascertains the health, finances, social life, and interpersonal relations of CGs, consisting of 22 items. Each item is scored with zero to four points; accordingly, the sum-score ranges from 0 (lowest burden) to 88 (highest burden). Three meaningful dimensions for clinicians can be established: social consequences for the CG, psychological burden, and feelings of guilt [52]. Initially, the ZBI was only used for CGs of patients with dementia, but it is now widely applied for different diseases [53,54,55]. As the cut-off for a higher risk of depression and anxiety is 24 points [55], we categorized the CGs for further analyses into two subgroups: a low-burden group (<24 points) and a high-burden group (≥24 points).

### 2.4. Statistical Analysis

The data management and all analyses were performed at Hannover Medical School. Statistical analysis was performed using IBM^®^ Statistical Software Package of Social Science (SPSS^®^, Chicago, IL, USA) version 26. Descriptive statistics were calculated and depicted as percentage, median, and range. In case of a missing item answer in any score, the individual arithmetic mean was applied. In case of more than one missing item, the datasets were excluded from the analysis. To test for normal distribution, the Shapiro–Wilk Test was performed, and as most of the data were not normally distributed, non-parametric tests were used. A marginal value of *p* ≤ 0.05 (two-tailed) was used for statistical interference for all analyses. We used a Mann–Whitney U test to compare two independent groups, a Kruskal–Wallis test to compare more than two independent groups for metric variables. For non-metric variables, a Fisher’s exact test was applied. Correlations were calculated by means of Spearman’s rank correlation coefficient. We did not perform a sample size calculation, and due to the exploratory character of the study, the results should not be regarded as confirmatory, but rather as hypothesis-generating.

Regression analysis was performed to measure the impact of demographics (age, sex, marital status), the patient’s disease characteristics (*SMN2* copy number, SMA type, scoliosis, vital capacity, HFMSE, RULM, ALSFRS-R, BI, ADI-12), and use of therapeutic aids (wheelchair, NIV, PEG) on HRQoL. First, a simple linear regression model was applied, with the EQ-5D-5L index value as dependent variable and the above-mentioned variables as independent variables. Subsequently, the variables with significant impact on HRQoL were included into a multiple linear regression model. Hereby, the EQ-5D-5L index value was the dependent variable, while the HFMSE was included as covariate in order to control for disease severity. The other scores measuring functional status/disease severity (RULM, ALSFRS-R, BI) and wheelchair use were not included in the multiple regression analysis, as they correlated strongly with the HFMSE (r_s_ > 0.8) and were identified as probable confounding variables. Backward elimination was performed to identify the variables with the highest impact (exclusion criterion = 0.1). Regression analysis to measure the impact of the assessed variables as mentioned above on CG burden (ZBI score) was performed in an analogous manner.

## 3. Results

### 3.1. HRQoL in SMA Patients

#### 3.1.1. Patients’ Characteristics

Two-thirds (66.7%) of the patients were diagnosed with SMA type III (including *n* = 1 type IV SMA patient) (median age 39 years) and 33.3% with SMA type II (median age 34 years). The study cohort mainly consisted of severely affected SMA patients using a wheelchair (65.4%), ventilatory support (23.1%) and PEG (7.7%). The detailed characteristics, median motor scores, and scores for ADL are depicted in Table 1.

The median EQ-5D-5L-VAS was 52.5, and the median EQ-5D-5L index value was 0.469, although patients formed two clusters: half of the patients reported high HRQoL (index value > 0.679) and the other half reported values <0.259. There were no readings in the range from 0.259 to 0.679. Regarding the SF-36, the lowest scores were achieved in the dimensions of Physical Functioning (median 5), Role Physical (median 50), General Health (median 52), and Vitality (median 55). The dimensions of Role Emotional and Bodily Pain, on the other hand, were mostly rated high (median 100) (Table 1). 

#### 3.1.2. Factors Associated with Patients’ HRQoL

Patients were clustered into either a high- or low-HRQoL group based on EQ-5D-5L index value scores. There were significant differences between the groups. Patients in the low-HRQoL group had a more severe phenotype (more frequently SMA type II, ≤3 *SMN2* copies, use of wheelchair, scoliosis, and lower RULM, HFMSE, ALSFRS-R, and BI) (Table 1). There were no significant differences between the groups regarding sex, BMI, marital status, use of NIV, or PEG and depression. Age, on the other hand, showed a difference between high- and low-HRQoL groups, with a median age of 39 in the high-HRQoL group vs. a median age of 33 in the low-HRQoL group (*p* = 0.028).

The EQ-5D-5L VAS correlated positively with RULM (*p* = 0.007; *n* = 22), HFMSE (*p* = 0.016; *n* = 22), and ALSFRS-R (*p* = 0.024; *n* = 22) (Appendix A).

**Figure 1:**
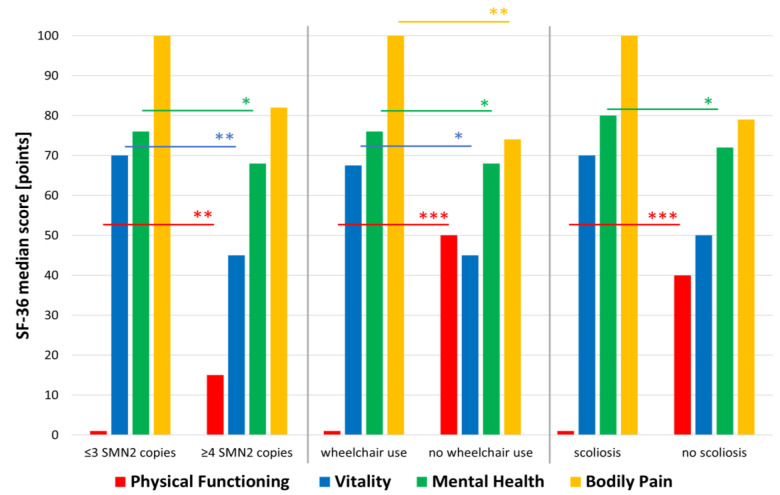
Association of the SF-36 dimensions Physical Functioning, Vitality, Mental Health, and Bodily Pain with patients’ characteristics. While patients with a more severe disease (≤3 *SMN2* copies, wheelchair use and scoliosis) reported low scores in the dimension of Physical Functioning, they reported better Mental Health and Vitality and less Bodily Pain. Abbreviations: *SMN2 = survival motor neuron 2* gene; SF-36 = Short Form Health survey 36; * *p* ≤ 0.05; ** *p* ≤ 0.01; *** *p* ≤ 0.001.

Regarding the SF-36, only Physical Functioning differed significantly between the two EQ-5D-5L index value groups (*p* = 0.004), meaning that both scales measured different constructs of HRQoL. Higher values in the SF-36 Physical Functioning dimension were consistently reported by SMA type III/IV patients (*p* < 0.001; *n* = 33), patients with ≥4 *SMN2* copies (*p* = 0.006; *n* = 27), and patients without NIV (*p* = 0.004; *n* = 33), wheelchair use (*p* < 0.001; *n* = 33), or scoliosis (*p* < 0.001; *n* = 32) (Figure 1). Physical Functioning further correlated positively with the HFMSE (*p* < 0.001; *n* = 33), RULM (*p* < 0.001; *n* = 33), ALSFRS-R (*p* < 0.001; *n* = 33), and BI (*p* = 0.001; *n* = 19) (Appendix A).

For the SF-36 dimension Vitality, significantly higher values were reported by males (*p* = 0.044; *n* = 32), participants with ≥12 educational years (*p* = 0.045; *n* = 29), and ≤3 *SMN2* copies (*p* = 0.002; *n* = 24). Furthermore, participants using a wheelchair reported a higher median than participants without wheelchair (*p* = 0.012; *n* = 30). Accordingly, Vitality correlated negatively with the RULM (*p* = 0.018; *n* = 30) and BI (*p* = 0.040; *n* = 19) (Appendix A).

While single participants reported a median of 100 in the dimension Bodily Pain, patients in a relationship reported significantly lower values (*p* = 0.041; *n* = 29). Similarly to the dimension Vitality, wheelchair use (*p* = 0.005; *n* = 29) as well as a lower RULM (*p* = 0.005; *n* = 29) and BI (*p* = 0.002; *n* = 19) were also associated with less Bodily Pain (Figure 1) (Appendix A).

Better Mental Health was associated with ≤3 *SMN2* copies (*p* = 0.029; *n* = 25), wheelchair use (*p* = 0.023; *n* = 31), and the presence of scoliosis (*p* = 0.031; *n* = 30), but not with motor function scores (RULM; HFMSE) or performance in ADL (ALSFRS-R, BI). Mental Health showed a strong negative correlation with the ADI-12 (*p* = 0.003; *n* = 31). The ADI-12 further correlated negatively with Vitality (*p* = 0.002; *n* = 30), Social Functioning (*p* = 0.043; *n* = 32), General Health (*p* = 0.038; *n* = 29), and Health Transition (*p* = 0.036; *n* = 33) (Appendix A).

#### 3.1.3. Regression Analysis of Predictors of Patients’ HRQoL

In the simple linear regression analyses, SMA type, *SMN2* copy number, wheelchair use, ALSFRS-R, RULM, HFMSE, NIV, and scoliosis had significant influences on HRQoL (EQ-5D-5L index value) (data not shown). Because predictors were highly correlated, in particular motor and ADL scores (HFMSE and RULM: *p* < 0.001, *n* = 31, Rho = 0.951; HFMSE and ALSFRS-R: *p* < 0.001, *n* = 39, Rho = 0.940; HFMSE and BI: *p* < 0.001, *n* = 22, Rho = 0.838), we excluded the RULM and the ALSFRS-R from the multiple regression analyses and kept the HFMSE as covariate for all analyses. Table 2 shows the results of the multiple linear regression analysis. Using backward selection, only HFMSE and NIV remained as significant influencing factors. The model including HFMSE, NIV, and scoliosis resulted in the best fit, explaining 82.8% of the variance (R² = 0.828, *p* < 0.001).

### 3.2. Caregiving in SMA

#### 3.2.1. Characteristics of Patient/Caregiver Pairs

Table 3 depicts the CGs’ and corresponding patients’ characteristics. Approximately three-quarters of the CGs were female with a median age of 52 years and were the primary CGs of their respective SMA patients. Most of the CGs were parents or spouses (each 32.7%) of the patients, and lived in the same household. The HRQoL of the CGs was high, with a median EQ-5D-5L VAS of 80/100 and a median EQ-5D-5L index value of 0.909. The median ZBI score reported by the CGs was 22/88, and the median duration of care (DOC) was 4.5 h per day, ranging from 0 to 22 h.

Regarding the patients’ characteristics in this study cohort, 61.2% were male with a median age of 29 years. Their median ALSFRS-R score was 30/48, and while 77.6% were dependent on a wheelchair, 26.5% of the patients used a PEG as well as NIV. Overall, the patients were more severely affected compared to the first study cohort. Patients’ HRQoL in this cohort was 60/100 (median EQ-5D-5L VAS) and 0.208 (median EQ-5D-5L index value) (Table 3).

#### 3.2.2. Factors Associated with CGB

To evaluate factors associated with CGB, we dichotomized the cohort into a low-burden group (median ZBI = 12.5/88, *n* = 28) and a high-burden group (median ZBI = 31/88, *n* = 21) using a score of 24 as a cut-off, as described previously [55]. Results are shown in Table 3.

The median DOC differed significantly between the low- (2.5 h) and high- (8.0 h) burden groups (*p* = 0.017). Further, though not statistically significant in the group comparison, a need of permanent attendance of a caregiver was associated with higher ZBI scores (median = 21 vs. median = 28.2). The relation to the patient seemed to be relevant for CG burden: while spouses were more often in the low-burden group (*n* = 14 vs. *n* = 2), children were more likely to be in the high-burden group (*n* = 11 vs. *n* = 3). Other characteristics of the CGs did not differ significantly between the groups.

Regarding the patients’ characteristics, age, and wheelchair use, BI and ALSFRS-R were associated with caregiver burden: the patients in the high-burden group were younger (*p* = 0.014), had a lower ALSFRS-R and BI (*p* = 0.018 and *p* = 0.008, respectively) and were more likely to use a wheelchair (*p* = 0.010) compared to the low-burden group (Table 3). This was supported by a negative correlation between the BI score, the ZBI score (s-rho = −0.556, *p* < 0.001, *n* = 49), and the DOC (s-rho = −0.626, *p* < 0.001, *n* = 49). Correspondingly, the ZBI and the DOC correlated negatively with the ALSFRS-R (*p* < 0.001; *n* = 49; s-Rho = −0.521; *p* < 0.001, *n* = 49, s-rho = −0.589).

**Figure 2:**
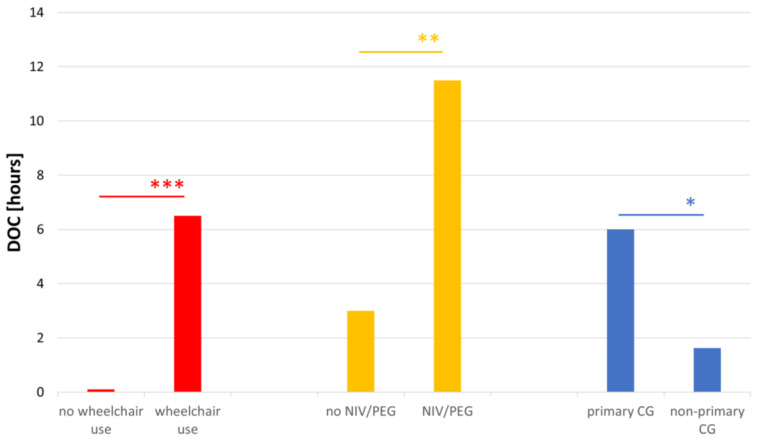
Association of the DOC with patients’ characteristics. Similarly to CGB, a higher DOC was associated with wheelchair use, as well as the use of NIV and PEG. Further, primary CGs had a higher DOC compared to non-primary CGs (*p* = 0.036, *n* = 49). Abbreviations: CG = caregiver; CGB = caregiver burden; DOC = duration of care; NIV = non-invasive ventilation; PEG = Percutaneous Endoscopic Gastrostomy; ZBI = Zarit Burden Interview * *p* ≤ 0.05; ** *p* ≤ 0.01; *** *p* ≤ 0.001.

A higher DOC was associated with wheelchair use (*p* < 0.001, *n* = 49; median = 0 h vs. median = 6.5 h) and the use of NIV and PEG (*p* = 0.002, *n* = 49; median = 3 h vs. median = 11.5 h). Further, the DOC was significantly higher for primary caregivers (median = 6.00, *n* = 37) compared to non-primary caregivers (median = 1.63, *n* = 11) (*p* = 0.036), as anticipated (Figure 2).

#### 3.2.3. Impact of CGB on Caregivers’ Health and HRQoL

Of the CGs, 46.9% stated that they had health impairments due to caregiving, all of them reporting physical impairments, such as back, muscle, knee, hip, and digestive pain. The majority of CGs who reported physical impairments (16 out of 23) additionally reported mental impairments, such as depression, burn-out, sleeping disorders, or anxiety. CGs who reported health impairments due to caregiving were more likely to be in the high-burden group (*p* = 0.017). Additionally, in the high-burden group there were more CGs who had physical and also mental impairments due to caregiving (*p* = 0.011) (Table 3).

The CGs’ median HADS-A and -D scores were 5/21 and 3/21, respectively. In the low-burden group, HADS-D and HADS-A were significantly lower compared to the high-burden group (*p* < 0.001 and *p* = 0.027, respectively). Nevertheless, all HADS results remained below 8, which has been described in the literature as the cut-off for clinically relevant anxiety and depression [50,51].

The HRQoL of CGs evaluated by means of the EQ-5D-5L was good in both the high- and low-burden groups. In contrast, several dimensions of the SF-36 differed significantly between the two groups: Vitality (50 vs. 65; *p* = 0.004), Bodily Pain (64 vs. 84; *p* = 0.021), Social Functioning (75 vs. 100; *p* = 0.001), and Mental Health (72 vs. 84; *p* = 0.003) were rated worse by CGs in the high-burden group.

#### 3.2.4. Regression Analysis of Predictors of CGB

First, we performed a simple linear regression to evaluate which parameters were associated with the ZBI score and therefore the CGB. Parameters with significant influence on the ZBI score were: the DOC, the necessity of permanent attendance of the CG, the CG’s age, the CG’s HADS-D and HADS-A, health impairments of the CG, the relationship between patient and CG, wheelchair use, use of NIV/PEG, the ALSFRS-R, and the BI (data not shown). Table 4 shows the results of the multiple linear regression analysis using a backward selection including all variables that were significant in the simple linear regression. The model maintaining BI, HADS-A, and health impairments of the CG resulted in the best fit, explaining 62.2% of the variance (R² = 0.622, *p* < 0.001) (Table 4).

## 4. Discussion

This multi-center study evaluated the HRQoL of adult SMA patients and their CGs as well as CGB using a number of PROMs. The main findings were correlations between the impairment of patients’ HRQoL and disease severity, and between CGs’ health and HRQoL and CGB.

The median EQ-5D-5L index value was 0.469 in the total SMA patient cohort, and 0.806 in the subgroup with higher HRQoL (which was nearly as high as the mean EQ-5D-5L index value in the general German population (0.880)) [56]. In the low-HRQoL group, however, the median EQ-5D-5L index value was 0.175, which was similar to findings from the United Kingdom (UK) (0.167), France (0.116), and Australia (0.115) for pediatric SMA patients [57,58]. Patients in the lower HRQoL subgroup more likely suffered from SMA type II and reported a more severe disease course. These characteristics are in line with previous studies that described an increase in the EQ-5D-5L from SMA type I to III along with a decreasing disease severity [4,57,59]. Median EQ-5D-5L VAS in our study cohort (52.5 points) was similar to previous studies [4,57,58,59,60].

Regarding the SF-36, the lowest scores were reached in the dimension Physical Functioning followed by Role Physical. This is in line with previous findings applying the SF-36 in NMDs [17,61]. A study from the Netherlands in adult SMA patients at an average age of 41.7 years showed the lowest scores in the dimension Physical Functioning [62], while a recent study conducted in a German SMA population found lower scores for Physical Functioning and General Health compared to healthy controls [14]. Patients with Duchenne muscular dystrophy (DMD) scored low in all dimensions except role limitations due to emotional problems (Role Emotional), with Physical Functioning, Role Physical, and Social Functioning being the most relevant dimensions [63,64]. In our cohort, Physical Functioning correlated positively with motor scores, and higher scores were associated with a milder phenotype, which is in line with the results of the above-mentioned studies [17,62,63].

Comparing the results of the two questionnaires, the EQ-5D-5L seems to incorporate mainly the physical aspects of HRQoL, which is corroborated by the strong correlation that we found for the EQ-5D-5L index value with the Physical Functioning dimension of the SF-36. The EQ-5D-5L primarily ascertains mobility, self-care, and usual activities—which all require a certain functional capability in the patient. The remaining two items of the EQ-5D-5L—pain/discomfort and anxiety/depression—have been shown to be less prevalent in SMA patients [61,65].

Interestingly, patients with more severe disease (SMA type II, lower motor scores), who were in the group with worse HRQoL according to the EQ-5D-5L index value, tended to have higher scores in the dimensions Mental Health, as well as Vitality and Bodily Pain as previously reported for Mental Health and Role Emotional [17,62]. SMA type III/IV patients, in contrast to SMA II, are usually older at disease onset and more frequently accustomed to a life without limitations in their daily activities, and thus find it more difficult to adapt to disease-related physical changes.

As expected, the mental aspects of HRQoL (Mental Health, Vitality, Social Functioning) and General Health correlated strongly with depressiveness when measured with the ADI-12. This was consistent with findings in ALS patients [66].

Our multiple linear regression model included the HFMSE as a substitute for the motor/functional scores. According to our model, a clinically significant increase of three points in the HFMSE would lead to an improvement of 0.039 points in the EQ-5D-5L index value. In contrast to findings in ALS [67,68,69], the use of NIV was found to be a negative predictor of HRQoL, most probably not due to the use of NIV itself but the underlying respiratory dysfunction together with more advanced disease as reported in DMD [70].

Our study not only showed a significant impact of SMA on patients themselves but also on their CGs. Similar to previous studies in SMA and other NMDs, the majority (77.6%) of the informal CGs in our cohort were female, and 65.4% were either the patients’ spouses/partners or their parents [71,72]. The median DOC in our cohort was 4.5 h per day, which is less than reported elsewhere in SMA caregiving [58,72]. This may be caused by the fact that in previous studies, pediatric SMA patients, who are usually more dependent on care due to their age, were the main population analyzed. DOC in a German ALS cohort was three hours per day, similarly to our findings [22]. The median ZBI score in our cohort was 22/88, and therefore below the cut-off of 24 points that defines a high burden [55]. As we stratified the CGs into low- and high-burden groups using this cut-off value, the CGs in the high-burden group had a median ZBI score of 31 points, and the corresponding patients were younger and suffered from more severe functional impairments (lower BI and ALSFRS-R scores). Accordingly, the DOC increased to eight hours per day in the high-burden group. A cross-sectional study conducted in Europe including the CGs of pediatric SMA patients similarly reported a mean ZBI score of 31.9/88 points [72]. Comparing our results with studies in adult ALS and DMD patients and their CGs, a higher burden (ZBI scores of 26/88 and 29/88 points, respectively) was estimated in these diseases [22,71,73]. Patients’ functional status (ALSFRS-R and BI scores) and the use of a wheelchair were associated with a higher CGB. We did not find a significant influence of NIV on CGB in our cohort, most likely due to the low number of patients using NIV. Further, the relationship between CG and patient significantly impacted the perceived burden, as spouses reported a lower burden than children. Similarly, a study from Brazil reported higher ZBI scores associated with a familial relationship to the patient [74].

In our study, nearly half of the CGs reported health impairments (46.9%), and one-third (32.7%) reported mental health impairments due to caregiving. Among the designated mental impairments, depression and anxiety were presented as main symptoms. However, the HADS-D and HADS-A median scores ranged below the cut-off for relevant depressive/anxious symptoms. Slightly higher scores were reported in a study from the Netherlands evaluating CGs of pediatric SMA patients with a mean HADS-D of 5.7 and a mean HADS-A of 6.8 points [75], with almost all the CGs being the patients’ mothers. On the one hand, the prevalence of depression and anxiety disorders is generally higher in women [76,77]; on the other hand, parents of severely impaired children may in general be more strained. Compared to the CGs of ALS patients, anxiety and depression scores in our CG cohort were lower, too, which could be due to slower disease progression, better prognosis, and more promising treatment options in SMA [73]. Focusing on the high-burden group, the HADS-D and HADS-A scores were similar to the above-mentioned studies. This emphasizes again the close interaction of the CGB and CG health. Whether depression and anxiety are consequences of the (high) CGB or CGs with depression/anxiety perceive the CGB to be higher cannot be conclusively determined within our study.

Multiple regression analysis revealed the BI, the HADS-A of the CG, and health impairments due to caregiving as main predictors of CGB. Similar findings were presented in studies with patients suffering from ALS, where mental health impairments in the CGs in particular, as well as wheelchair use and the need for permanent supervision of the patient, led to a significant increase in the ZBI score [22,49,73]. This highlights the importance not only of the functional impairments of the patient as a predictor for CGB, but also of the CGs’ (mental) health, which seems to interact closely with perceived burden in MNDs [49,73,78].

Regarding the HRQoL of SMA CGs, median EQ-5D-5L VAS (80) and index value (0.910) were similar to previously reported values for CGs of SMA patients from the UK (VAS 80.36) and Canada (VAS 81.3) [58,59]. Overall, in this study the HRQoL of the CGs was similar to that of the general population in Germany [56]. This is in line with findings for CGs in ALS, who also reported good HRQoL with a median EQ-5D-5L VAS of 75 and an index value of 0.909 [22,73]. In the absence of data in SMA, we compared our results regarding the dimensions of HRQoL (SF-36) to reports in other NMDs. A study from Korea in ALS patients in an advanced disease stage and a mean patient age of 52.6 years reported low scores for the primary CGs throughout all dimensions of the SF-36 [79]. In our study, SF-36 scores approximated to the general German population [80]. One reason for the comparably high scores in our study could be the planned or ongoing disease-modifying treatment with nusinersen [81]. In contrast, patients suffering from ALS have a worse prognosis and mainly receive symptomatic treatment. Further, CGs in the high-burden group presented with lower scores in the dimensions of Bodily Pain, Vitality, Social Functioning, and Mental Health. These findings point towards the importance of mental factors involved in and psychological consequences associated with perceived CGB.

Overall, the good HRQoL of SMA CGs contrasts with the high perceived burden and the reported health impairments.

One advantage of our study was the variety of factors possibly influencing HRQoL and CG burden that were analyzed, such as demographic and the clinical and psychological characteristics of patients and CGs. Further, we intended to depict an overall picture of the impact of SMA on patients’ and CGs’ health by analyzing not only the patients’ functional status, HRQoL, and depression, but also the CGs’ burden, health impairments and HRQoL. Among the study’s limitations, the low participant number must be mentioned. Due to the rarity of SMA, larger cohorts are difficult to acquire, especially considering that for the evaluation of CGB, not only the patient but also the CG needs to consent to study participation. Nevertheless, our study was a multi-center study representing five specialized motor neuron disease clinics throughout Germany. We captured patients’ HRQoL in an overlapping but finally different cohort from the patient/CG pairs included in the analysis of CGB, making direct correlations between the HRQoL of patients and CGs or the CGB impossible. Moreover, a longitudinal evaluation of HRQoL and CGB would have been desirable to quantify the effects of nusinersen treatment on non-motor outcomes. Generally, the instruments we used to evaluate the HRQoL of SMA patients as well as CGB must be discussed. Both the EQ-5D-5L and SF-36 as well as the ZBI are generic instruments initially developed for use in other diseases [41,46,52]. Questionnaires capturing aspects specific to NMDs [29] may have yielded more reliable and representative data on the HRQoL in the analyzed SMA cohort. However, using the EQ-5D-5L and the SF-36, which are widely used in various diseases, facilitates comparison with other diseases. Landfeldt et al. found that the ZBI is “not fit for purpose to measure burden in caregivers of patients with DMD” [71]. Like DMD, SMA is a progressively disabling NMD, with childhood onset leading to dependency and need for care. Accordingly, it is possible that the ZBI does not represent CGB in SMA properly. In the same manner, the ADI-12 might not adequately capture SMA patients’ depression, even though it is validated for ALS.

Regarding bias and imprecision, we have to consider a possible selection bias, as all participants were recruited at specialized motor neuron centers and therefore possibly less severely affected than SMA patients who do not visit specialized clinics. Lastly, all data were acquired during ongoing nusinersen treatment, which could have led to higher HRQoL scores due to the participants’ positive expectations regarding treatment effects. Furthermore, the presented results might be true for the adult German SMA population, as participant characteristics are congruent with other reports [36,82]. However, in pediatric SMA populations or in non-industrialized countries, the situation might be different.

Our findings add to the emerging research on PROM for the evaluation of novel treatment options in SMA [14,15,58,60,62,82,83,84]. This study confirms that HRQoL in SMA patients is impaired with regard to physical aspects and determined by disease severity. Disease severity is also the main predictor of the CGB, which itself affects the CGs’ health and HRQoL. This close and reciprocal interaction between the (physical) well-being of the patient and the well-being of the respective CG highlights the need not only to focus on the patients’ physical abilities but rather grasp at an overall picture of patient and CG health while evaluating novel treatments. Along with the novel treatment options—in particular, nusinersen as intrathecal ASO, intravenous gene therapy with onasemnogene abeparvovac, and the oral splicing modifier risdiplam [9,10,85,86,87,88]—the SMA phenotype is expected to evolve. Pediatric patients with SMA type I and II will reach adulthood more frequently, severe motor impairments will hopefully be less common, and new (possibly treatment-associated) difficulties will surface. In this context, requirements for the care and follow-up of SMA patients will change, making PROM such as HRQoL and CGB valuable tools to assess the efficacy and secondary effects of the new treatments. Future studies should focus on the validation of disease-specific or generic tools to assess HRQoL and CGB in SMA. Furthermore, exploratory studies should refine what kind of supportive measures are needed to relieve the burden of CGs of SMA patients.

## 5. Conclusions

In conclusion, in SMA, not only the patient’s HRQoL is drastically impaired, but the CG’s health can also be affected due to high CGB. To improve both the patient and CG well-being, physical as well as mental health aspects must be taken into account, which will become all the more relevant in light of the novel treatments and changing SMA phenotypes.

## Figures and Tables

**Table 1:** Patients’ characteristics and HRQoL: This table shows the demographic and clinical characteristics of all patients participating in the HRQoL analysis. The patients were dichotomized into high- and low-HRQoL groups according to their EQ-5D-5L index value (>0.679 or <0.259). Statistical parameters printed in bold type indicate statistically significant differences between the high- and low-HRQoL groups. * indicates that a Mann–Whitney U Test was performed. # indicates that either chi-Square or Fisher’s exact tests were performed. Abbreviations: EQ-5D-5L = EuroQoL Five Dimension Five Level Scale; HRQoL = health-related quality of life; *n* = number; BMI = Body Mass Index; SMA = spinal muscular atrophy; *SMN2 = survival motor neuron* 2 gene; NIV = non-invasive ventilation; PEG = percutaneous endoscopic gastrostomy; ALSFRS-R = Amyotrophic Lateral Sclerosis Functional Rating Scale Revised; RULM = Revised Upper Limb Module; HFMSE = Hammersmith Functional Motor Scale Expanded; ADI-12 = ALS-Depression Inventory-12; VAS = Visual Analogue Scale; SF-36 = Short Form Health Survey 36.

Patients’ Characteristics and HRQoL Patients’ Characteristics	All *n* (%)/Median (Range)	EQ-5D-5L Index Value > 0.679 *n* = 11 (100%)	EQ-5D-5L Index Value < 0.259 *n* = 11 (100%)	
Sex (*n* = 39) Male Female	25 (64.1%) 14 (35.9%)	5 (45.5%) 6 (54.5%)	8 (72.7%) 3 (27.3%)	*p* = 0.193; *n* = 22 #
Age, years (*n* = 39)	36 (19–65)	39 (22–64)	33 (19–51)	*p* = 0.028, *n* = 22 *
BMI (kg/m²) (*n* = 30)	22.9 (10.7–35.9)	22.9 (18.6–32.3)	22.9 (10.7–35.9)	*p* = 0.478, *n* = 22 *
Marital status (*n* = 39) Single Married/in a relationship	27 (69.2%) 12 (30.8%)	6 (54.5%) 5 (45.5%)	9 (81.8%) 2 (18.2%)	*p* = 0.170; *n* = 22 #
SMA type (*n* = 39) II III/IV	13 (33.3%) 26 (66.7%)	1 (9.1%) 10 (90.9%)	7 (63.6%) 4 (36.4%)	***p* = 0.008; *n* = 22 #**
*SMN2* copy number (*n* = 39) ≤3 ≥4 Unknown	17 (43.6%) 16 (41%) 6 (15.4%)	2 (18.2%) 9 (81.8%)	9 (81.8%) 2 (18.2%)	***p* = 0.003; *n* = 22 #**
Use of wheelchair (*n* = 39) Yes No	22 (56.4%) 17 (43.6%)	1 (9.1%) 10 (90.9%)	11 (100%) 0 (0%)	***p* < 0.001; *n* = 22 #**
Use of NIV (*n* = 39) Yes No	9 (23.1%) 30 (76.9%)	0 (0%) 11 (100%)	4 (36.4%) 7 (63.6%)	*p* = 0.027; *n* = 22 #
Use of PEG (*n* = 39) Yes No	3 (7.7%) 36 (92.3%)	0 (0%) 11 (100%)	1 (9.1%) 10 (90.9%)	*p* = 0.306; *n* = 22 #
Scoliosis (*n* = 39) Yes No Unknown	17 (43.6%) 21 (53.8%) 1 (2.6%)	2 (18.2%) 9 (81.8%)	8 (72.7%) 2 (18.2%) 1 (9.1%)	***p* = 0.005; *n* = 22 #**
Barthel-Index (*n* = 22); 0–100	47.5 (10–100)	95 (80–100)	25 (10–35)	*p* = 0.036, *n* = 8 *
ALSFRS-R score (*n* = 39); 0–48	34 (0–48)	43 (29–48)	29 (10–38)	*p* < 0.001, *n* = 22 *
RULM (*n* = 39); 0–37	21 (0–37)	37 (7–37)	12 (0–30)	*p* = 0.002, *n* = 22 *
HFMSE (*n* = 39); 0–66	12 (0–66)	47 (2–63)	6 (0–19)	*p* < 0.001, *n* = 22 *
ADI-12 (*n* = 35); 0–48	18 (12–32)	20 (14–30)	16.5 (12–32)	*p* = 0.314, *n* = 21 *
**Measures of HRQoL** **EQ-5D-5L**	**All** ***n* (%)/Median (range)**	**EQ-5D-5L Index Value > 0.679** ***n* = 11 (100%)**	**EQ-5D-5L Index Value** **< 0.259** ***n* = 11 (100%)**	
EQ-5D-5L VAS (*n* = 22); 0–100	52.5 (25–100)	60 (35–75)	50 (25–100)	*p* = 0.065, *n* = 22 *
EQ-5D-5L Index Value (*n* = 22); −0.661–1	0.469 (0.031–0.918)	0.806 (0.679–0.918)	0.175(0.031–0.259)	
**SF-36; 0–100**				
Physical Functioning (*n* = 33)	5 (0–90)	15 (0–75)	0 (0–10)	*p* = 0.004, *n* = 21 *
Role Physical (*n* = 34)	50 (0–100)	50 (0–100)	25 (0–100)	*p* = 0.401, *n* = 22 *
Role Emotional (*n* = 30)	100 (0–100)	100 (0–100)	100 (100)	*p* = 0.515, *n* = 18 *
Vitality (*n* = 30)	55 (15–100)	45 (20–65)	65 (15–100)	*p* = 0.146, *n* = 18 *
Mental Health (*n* = 31)	72 (52–100)	68 (52–80)	76 (52–100)	*p* = 0.156, *n* = 19 *
Social Functioning (*n* = 32)	75 (25–100)	75 (25–100)	75 (25–100)	*p* = 1.000, *n* = 20 *
Bodily Pain (*n* = 29)	100 (41–100)	74 (51–100)	100 (41–100)	*p* = 0.364, *n* = 17 *
General Health (*n* = 29)	52 (17–87)	52 (40–70)	40 (35–72)	*p* = 0.364, *n* = 17 *
Health Transition (*n* = 34)	50 (25–100)	25 (25–50)	50 (25–50)	*p* = 0.300, *n* = 22 *

**Table 2:** Influencing factors on HRQoL in SMA patients during nusinersen-loading period. The EQ-5D-5L index value was the dependent variable in the multiple linear regression analysis with backward stepwise selection. The HFMSE was used as covariate. Abbreviations: CI = confidence interval, HFMSE = Hammersmith Functional Motor Scale Expanded, NIV = non-invasive ventilation.

Parameter	Regression Coefficient	Standardized Coefficient (*ß*)	Std. Error	*p*-Value	95% CI
HFMSE	0.013	1.019	0.003	0.002	0.006–0.020
NIV	−0.266	−0.375	0.114	0.047	−0.529–−0.004
Scoliosis	0.307	0.453	0.155	0.083	0.665–0.300

**Table 3:** Caregivers’ characteristics, burden and HRQoL. This table shows the demographic and clinical characteristics of the patient/caregiver pairs. The pairs were dichotomized into a high-burden group and a low-burden group according to the CG’s ZBI score (cut-off = 24). Statistical parameters printed in bold type indicate statistically significant differences between the high- and the low-burden groups. * indicates that a Mann–Whitney U Test was performed. # indicates that either chi-Square or Fisher’s exact tests were performed. Abbreviations: *n* = number; HRQoL = health-related quality of life; CG = caregiver; ZBI = Zarit Burden Interview; DOC = duration of care; HADS-D = Hospital Anxiety and Depression Scale—Depression Subscale; HADS-A = Hospital Anxiety and Depression Scale—Anxiety Subscale; EQ-5D-5L = EuroQoLFive Dimension Five Level Scale; VAS = Visual Analog Scale; SF-36 = Short Form Health Survey 36; ALSFRS-R = Amyotrophic Lateral Sclerosis Functional Rating Scale Revised; PEG= percutaneous endoscopic gastrostomy; NIV = non-invasive ventilation.

Caregivers’ Characteristics, Burden, and HRQoL Caregivers’ Characteristics	All *n* (%)/Median (Range)	Low Burden ZBI < 24 *n* = 28 (100%)/ Median (Range)	High Burden ZBI ≥ 24 *n* = 21 (100%)/ Median (Range)	
Sex (*n* = 49) Male Female	11 (22.4%) 38 (77.6%)	8 (28.6%) 20 (71.4%)	3 (14.3) 18 (85.7%)	*p* = 0.236; *n* = 49 #
Age, years (*n* = 47)	52 (24–77)	52 (24–77)	51.5 (41–72)	*p* = 0.282; *n* = 47 *
Marital status (*n* = 49) Single Married/partner	6 (12.2%) 43 (87.7%)	3 (10.7%) 25 (89.3%)	3 (14.3%) 18 (85.7%)	*p* = 0.706; *n* = 49 #
Primary caregiver (*n* = 49) Yes No	37 (75.5%) 12 (24.5%)	20 (71.4%) 8 (28.6%)	17 (81%) 4 (19%)	*p* = 0.443; *n* = 49 #
Permanent CG attendance necessary (*n* = 25) Yes No	12 (48.0%) 13 (52.0%)	4 (30.8%) 9 (69.2%)	8 (66.7%) 4 (33.3%)	*p* = 0.073; *n* = 25 #
Relation to the patient (*n* = 49) Spouse Parent Sibling Child Other	16 (32.7%) 16 (32.7%) 2 (4.1%) 14 (28.6%) 1 (2%)	14 (50%) 9 (32.1%) 1 (3.6%) 3 (10.7%) 1 (3.6%)	2 (9.5%) 7 (33.3%) 1 (4.8%) 11 (52.4%) 0 (0%)	***p* = 0.007; *n* = 49 #**
Employment (*n* = 49) Working Not working Retired or homemaker	28 (57.1%) 7 (14.3%) 14 (28.6%)	18 (64.3%) 2 (7.1%) 8 (28.6%)	10 (47.6%) 5 (23.8%) 6 (28.6%)	*p* = 0.233; *n* = 49 #
**Caregivers’ burden, health impairments, and HRQoL**	**All** ***n* (%)/Median (Range)**	**Low Burden ZBI < 24** ***n* = 28 (100%)/** **Median (Range)**	**High Burden ZBI ≥ 24** ***n* = 21 (100%)/** **Median (Range)**	
DOC, hours (*n* = 49)	4.5 (0–22)	2.5 (0–22)	8.0 (0–22)	*p* = 0.017, *n* = 49 *
ZBI score (*n* = 49); 0–88	22 (0–42)	12.5 (0–23)	31 (25–42)	
Health impairment due to caregiving (*n* = 49) Yes No	23 (46.9%) 26 (53.1%)	9 (32.1%) 19 (67.9%)	14 (66.7%) 7 (33.3%)	***p* = 0.017; *n* = 49 #**
Physical and mental health impairments due to caregiving (*n* = 49)	16 (32.7%)	5 (17.9%)	11 (52.4%)	***p* = 0.011; *n* = 23 #**
HADS-D score (*n* = 45); 0–21	3 (0–11)	2 (0–7)	6 (1–11)	*p* < 0.001, *n* = 45 *
HADS-A score (*n* = 45); 0–21	5 (0–12)	4.5 (0–9)	6 (1–12)	*p* = 0.027, *n* = 45 *
**EQ-5D-5L**				
EQ-5D-5L VAS (*n* = 47); 0–100	80 (40–100)	80 (60–100)	80 (40–90)	*p* = 0.115, *n* = 47 *
EQ-5D-5L Index Value (*n* = 48); −0.661–1	0.910 (0.085–1)	0.910 (0.085–1.000)	0.909 (0.291–1.000)	*p* = 0.041, *n* = 48 *
**SF-36; 0–100**				
Physical Functioning (*n* = 49)	95 (0–100)	95 (0–100)	90 (70–100)	*p* = 0.623, *n* = 49 *
Bodily Pain (*n* = 49)	74 (22–100)	84 (22–100)	64 (22–100)	*p* = 0.021, *n* = 49 *
General Health (*n* = 49)	72 (37–100)	74.5 (37–100)	67 (45–97)	*p* = 0.366, *n* = 49 *
Vitality (*n* = 49)	60 (10–85)	65 (25–80)	50 (10–85)	*p* = 0.004, *n* = 49 *
Social Functioning (*n* = 49)	87.5 (38–100)	100 (63–100)	75 (38–100)	*p* = 0.001, *n* = 49 *
Role Emotional (*n* = 49)	100 (0–100)	100 (0–100)	100 (0–100)	*p* = 0.413, *n* = 49 *
Mental Health (*n* = 49)	76 (28–92)	84 (36–92)	72 (28–84)	*p* = 0.003, *n* = 49 *
Health Transition (*n* = 49)	50 (25–100)	50 (25–100)	50 (25–100)	*p* = 0.151, *n* = 49 *
**Patients’ characteristics**	**All** ***n* (%)/Median (Range)**	**Low Burden ZBI < 24** ***n* = 28 (100%)/Median (Range)**	**High Burden ZBI ≥ 24** ***n* = 21 (100%)/** **Median (Range)**	
Sex (*n* = 49) Female Male	19 (38.8%) 30 (61.2%)	9 (32.1%) 19 (67.9%)	10 (47.6%) 11 (52.4%)	*p* = 0.271; *n* = 49 #
Age (*n* = 49)	29 (7–65)	32 (7–65)	24 (11–49)	*p* = 0.014, *n* = 49 *
Wheelchair use (*n* = 49) Yes No	38 (77.6%) 11 (22.4%)	18 (64.3%) 10 (35.7%)	20 (95.2%) 1 (4.8%)	***p* = 0.010; *n* = 49 #**
NIV and PEG (*n* = 49) Yes No	13 (26.5%) 36 (73.5%)	5 (17.9%) 23 (82.1%)	8 (38.1%) 13 (61.9%)	*p* = 0.112; *n* = 49 #
Barthel-Index (*n* = 49)	30 (0–100)	35 (0–100)	25 (0–95)	*p* = 0.008, *n* = 49 *
ALSFRS-R score (*n* = 49); 0–48	30 (0–47)	33 (0–47)	28 (16–39)	*p* = 0.018, *n* = 49 *
EQ-5D-5L VAS (*n* = 28); 0–100	60 (10–100)	65 (10–90)	55 (30–100)	*p* = 0.387, *n* = 28 *
EQ-5D-5L Index Value (*n* = 28); −0.661–1	0.208 (−0.018–1)	0.175 (−0.018–1)	0.241 (0.063–0.755)	*p* = 0.457, *n* = 28 *

**Table 4:** Regression analyses of predictors of CGB: The ZBI score was the dependent variable in the multiple linear regression with backward stepwise selection. Abbreviations: HADS-A = Hospital Anxiety and Depression Scale–Anxiety Subscale, CI = confidence interval.

Parameter	Regression Coefficient	Standardized Coefficient (*ß*)	Std. Error	*p*-Value	95% CI
Barthel-Index	−0.137	−0.424	0.046	0.009	−0.235–−0.039
HADS-A	1.447	0.403	0.510	0.011	0.372–2.523
Health impairments due to caregiving	8.873	0.396	3.312	0.016	1.885–15.860

## Data Availability

All data generated or analyzed during this study are included in this published article and will be shared with any qualified researcher on reasonable request.

## Appendix A

**Table A1:** Correlation analyses of HRQoL: Motor/ADL scores correlated positively with EQ-5D-5L and Physical Functioning, while Bodily Pain, Vitality, and Role Emotional were negatively correlated with them. The ADI-12 correlated negatively with Vitality, Mental Health, General Health, and Social Functioning. Abbreviations: ADL = activities of daily living; ADI-12 = ALS-Depression Inventory-12; ALSFRS-R = Amyotrophic Lateral Sclerosis Functional Rating Scale Revised; BI = Barthel Index; EQ-5D-5L = Euro QoL Five Dimension Five Level Scale; HFMSE = Hammersmith Functional Motor Scale Expanded; HRQoL = health-related quality of life; RULM = Revised Upper Limb Module; SF-36 = Short Form Health Survey 36; VAS = Visual Analogue Scale.

	HFMSE	RULM	ALSFRS-R	BI	ADI-12
EQ-5D-5L Index Value	*p* < 0.001; *n* = 22; S-Rho = 0.805	*p* = 0.001; *n* = 22; S-Rho = 0.659	*p* < 0.001; *n* = 22; S-Rho = 0.789		
EQ-5D-5L VAS	*p* = 0.016; *n* = 22; S-Rho = 0.507	*p* = 0.007; *n* = 22; S-Rho = 0.559	*p* = 0.024; *n* = 22; S-Rho = 0.478		
SF-36 Physical Functioning	*p* < 0.001; *n* = 33; S-Rho = 0.886	*p* < 0.001; *n* = 33; S-Rho = 0.828	*p* < 0.001; *n* = 33; S-Rho = 0.908	*p* = 0.001; *n* = 19; S-Rho = 0.874	
SF-36 Role Physical					
SF-36 Social Functioning					*p* = 0.043; *n* = 32; S-Rho = −0.361
SF-36 Role Emotional				*p* = 0.04; *n* = 18; S-Rho = −0.487	
SF-36 Vitality		*p* = 0.018; *n* = 30; S-Rho = −0.430	*p* = 0.027; *n* = 30; S-Rho = −0.403	*p* = 0.04; *n* = 19; S-Rho = −0.474	*p* = 0.002; *n* = 30; S-Rho = −0.553
SF-36 Bodily Pain	*p* = 0.012; *n* = 29; S-Rho = −0.462	*p* = 0.005; *n* = 29; S-Rho = −0.505	*p* = 0.003; *n* = 29; S-Rho = −0.532	*p* = 0.002; *n* = 19; S-Rho = −0.654	
SF-36 Mental Health					*p* = 0.003; *n* = 31; S-Rho = −0.513
SF-36 General Health					*p* = 0.038; *n* = 29; S-Rho = −0.388
SF-36 Health Transition					*p* = 0.036; *n* = 33; S-Rho = −0.366
